# Case Report: virus infection-induced exacerbations of lung injury and fibrosis in children with diffuse alveolar hemorrhage: report of three cases

**DOI:** 10.3389/fped.2025.1590800

**Published:** 2025-08-21

**Authors:** Qing Wei, Jing Liu, Xun Chen, Chunyan Li, Guangmin Nong, Yan Li

**Affiliations:** ^1^ Department of Pediatrics, The First Affiliated Hospital of Guangxi Medical University, Nanning, Guangxi, China; ^2^ Department of Radiology, The First Affiliated Hospital of Guangxi Medical University, Nanning, Guangxi, China

**Keywords:** virus infection, lung injury and fibrosis, diffuse alveolar hemorrhage, ground glass opacity, interlobular septal thickening

## Abstract

Virus infection is a common cause of lung injury and can result in lung fibrosis in severe cases. Furthermore, it is a significant trigger of disease exacerbation in patients with lung fibrosis. However, nearly all the case reports or case series to date have focused on the adult population rather than the pediatric population. Here, we report three cases of virus infection-induced exacerbations of lung injury and fibrosis in children with diffuse alveolar hemorrhage. To our knowledge, this is one of the first articles to report virus infection-induced lung injury and fibrosis in children.

## Introduction

Virus infection is a common cause of lung injury and can result in lung fibrosis in severe cases (1). Furthermore, it is a significant trigger of disease exacerbations in patients with lung fibrosis (1). Diffuse alveolar hemorrhage (DAH) is a severe, life-threatening clinical syndrome classified within the group of interstitial lung diseases (ILD) (2). It is characterized by the accumulation of red blood cells in the alveolar spaces (2). In the end stage of the disease, recurrent episodes of alveolar hemorrhage can lead to the development of lung fibrosis (2). In clinical practice, episodes of alveolar hemorrhage have been observed to be induced by virus infection in the DAH, which has attracted certain attention (3, 4). However, virus infection-induced exacerbations of lung injury and fibrosis have not yet attracted enough attention. Here, we report three cases of virus infection-induced exacerbations of lung injury and fibrosis in children with DAH with the aim of extending clinician' awareness of this disease. All three cases presented with a chronic, but relatively stable clinical course; however, exacerbations of lung injury and fibrosis developed after virus infection. To our knowledge, this is one of the first articles to report virus infection-induced lung injury and fibrosis in children.

## Case report

### Case presentation 1

A 12-year-old boy, born to non-consanguineous parents, presented with a 1-day history of fever, fatigue, cough, nasal congestion, and rhinorrhea. He required continuous oxygen inhalation upon admission. He had repeated anemia and intermittent hemoptysis since the age of 4 and was diagnosed with DAH. He had been treated with glucocorticoids and immunosuppressants for 8 years. Despite treatment, the disease easily relapsed and needed high-dose glucocorticoid treatment. Before this exacerbation, the case presented with a chronic, but relatively stable clinical course.

**Figure 1:**
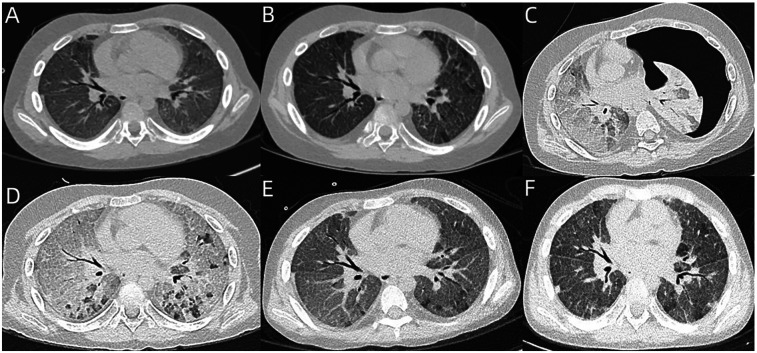
**(A)** Chest CT of case 1 revealed mild GGO and scattered interlobular septal thickening. **(B)** Reexamined chest CT after 2 weeks revealed partial resolution of GGO, but no improvement of interlobular septal thickening. **(C,D)** Chest CT revealed severe diffuse GGO, significantly increased interlobular septal thickening, and left-sided massive pneumothorax. After treatment, the pneumothorax had completely resolved, whereas severe diffuse GGO and interlobular septal thickening still remained in both lungs. **(E,F)** Follow-up chest CT at 5 months and 6 months revealed partial resolution of GGO and interlobular septal thickening.

On examination, he exhibited shortness of breath and suprasternal retraction, but no lung rales. Laboratory investigations showed mild anemia, neutrophilic leukocytosis, mild procalcitonin (PCT) elevation, hypoxemia, and a normal C-reactive protein (CRP) level. Targeted next-generation sequencing (tNGS) gene panels for respiratory infection using the nasopharyngeal swabs showed a high sequence number (reads = 59,806) of influenza A H1N1 virus (FluA/H1N1). Chest computed tomography (CT) revealed mild diffuse ground glass opacity (GGO) and scattered interlobular septal thickening (Figure 1A). Bronchoscopy revealed dark red-brown bronchoalveolar lavage fluid (BALF), and tNGS gene panels for respiratory infection using BALF showed a high sequence number (reads = 7,570) of FluA/H1N1.

Oral single-dose baloxavir marboxil (40 mg) was administered to treat influenza timely, and intravenous methylprednisolone (2 mg/kg/day) was administered to treat DAH. After 2 weeks, his dyspnea had only slightly improved. A follow-up chest CT revealed partial resolution of GGO, but no improvement of interlobular septal thickening (Figure 1B). During the next 2 weeks, his dyspnea had no improvement, and he continued to require continuous oxygen inhalation. BALF reexamination presented as rust-colored. Notably, the Hb level reexamination was normal. As he had a sudden worsening of dyspnea, chest CT was reexamined and revealed severe diffuse GGO, significantly increased interlobular septal thickening, and left-sided massive pneumothorax (Figure 1C). The pneumothorax had completely resolved after treatment with closed thoracic drainage, whereas severe diffuse GGO and interlobular septal thickening still remained (Figure 1D). Virus infection-induced exacerbations of lung injury and fibrosis were considered, and nintedanib was administered. His dyspnea gradually improved, but he still required intermittent oxygen inhalation after 2 months of nintedanib treatment. Thereafter, nintedanib was withdrawn due to the social and personal reasons of the case. However, oxygen inhalation was withdrawn successfully 3 months later. The follow-up chest CT revealed partial resolution of GGO and interlobular septal thickening (Figures 1E,F).

### Case presentation 2

A 7-year-old girl, born to non-consanguineous parents, presented with a 4-week history of dyspnea and cough and required continuous oxygen inhalation. She had a long-standing history of recurrent anemia, with no identifiable cause. She was diagnosed with DAH and received the treatment of glucocorticoids 1 year ago. However, the disease easily relapsed. Before this exacerbation, the case also presented with a chronic, but relatively stable clinical course.

**Figure 2:**
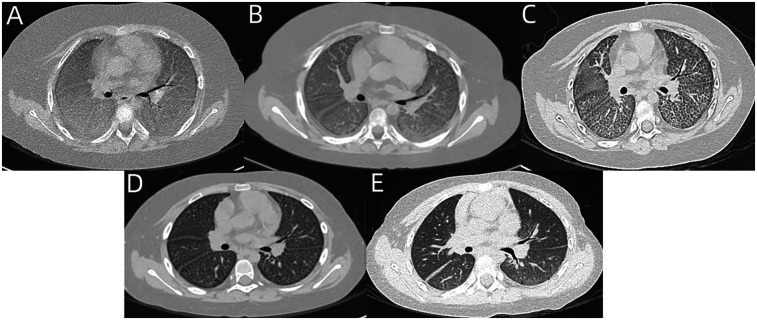
**(A)** Chest CT of case 2 revealed diffuse GGO and marked interlobular septal thickening. **(B–E)** Follow-up chest CT at 1 month, 3 months, 6 months, and 1 year revealed partial resolution of GGO and interlobular septal thickening.

On examination, she exhibited shortness of breath, suprasternal retraction, and moist rales at end-inspiration. Laboratory investigations showed hypoxemia, but no anemia, and normal white blood cell (WBC), CRP, and PCT levels. Chest CT revealed diffuse GGO, marked interlobular septal thickening (Figure 2A). BALF presented as cloudy and white. tNGS gene panels for respiratory infection using BALF only showed cytomegalovirus (CMV) (reads = 735). Virus infection-induced exacerbations of lung injury and fibrosis were considered. Therefore, ganciclovir was administered intravenously. Two weeks later, reexamination of tNGS gene panels for respiratory infection using BALF showed decreased sequences of CMV (reads = 60). Ganciclovir intravenously was administered for another 2 weeks. Six months later, reexamination of the tNGS gene panels for respiratory infection using BALF showed the absence of CMV. The follow-up chest CT revealed partial resolution of GGO and interlobular septal thickening (Figures 2B–E).

### Case presentation 3

**Figure 3:**
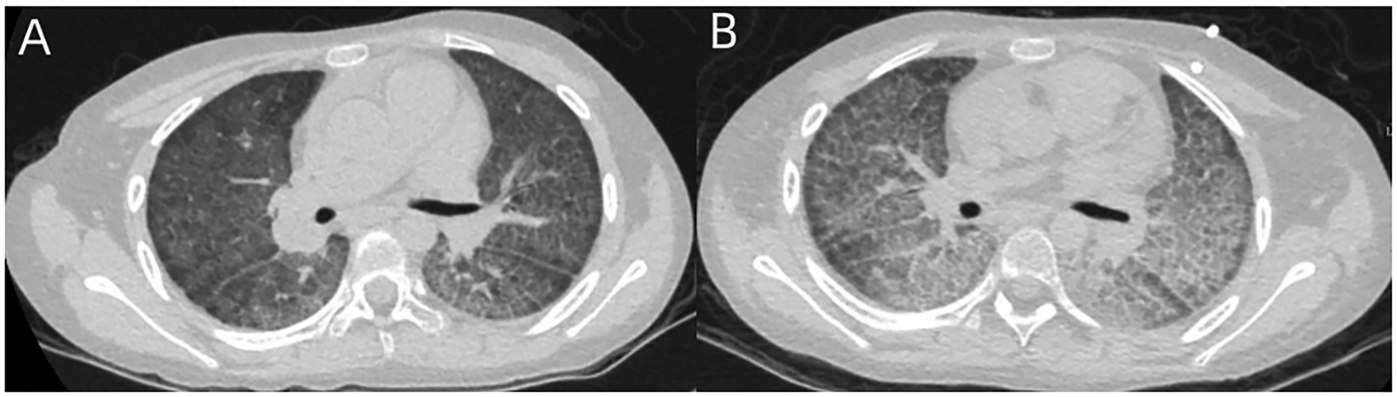
**(A)** Chest CT of case 3 revealed diffuse GGO and marked interlobular septal thickening. **(B)** The follow-up chest CT at 4 weeks still revealed severe diffuse GGO and interlobular septal thickening.

A 9-year-old girl, born to non-consanguineous parents, had a history of recurrent anemia and intermittent hemoptysis since the age of 6. Both chest CT and BALF analysis for the detection of hemosiderophages had been performed. Ultimately, she had been diagnosed with DAH and began to receive the treatment of glucocorticoids 2 months ago. After the treatment, the patient presented with a relatively stable clinical course. Subsequently, she presented with a 2-day history of fever, fatigue, cough, nasal congestion, and rhinorrhea due to human parainfluenza virus (HPIV) infection, as the tNGS gene panels for respiratory infection using the nasopharyngeal swabs showed a high sequence number (reads = 41,766). As no effective antivirus drug for HPIV was available, she was treated with symptomatic treatment and supportive care. Chest CT revealed diffuse GGO and marked interlobular septal thickening (Figure 3A). Thereafter, she developed worsening dyspnea and required continuous oxygen inhalation. The follow-up chest CT at 4 weeks after HPIV infection still revealed severe diffuse GGO and interlobular septal thickening (Figure 3B). Virus infection-induced exacerbations of lung injury and fibrosis were considered. Also, she developed an episode of alveolar hemorrhage as the Hb level decreased significantly. Unfortunately, she died due to bacterial and fungal co-infection 4 weeks later, though she had received the appropriate antibacterial as well as antifungal drugs.

## Discussion

Viruses are one of the major pathogens of pneumonia. Usually, viral pneumonia is self-limiting and resolves completely. However, in some cases, it may develop into diffuse alveolar injury, which can result in fibrosis or even death (1, 5, 6). Currently, virus infection-induced lung injury and fibrosis have been gaining increasing attention(1). Notably, nearly all the case reports or case series focused on the adult population rather than the pediatric population. In children, postinfectious bronchiolitis obliterans (BO) caused by the virus, especially adenovirus infection, has received certain attention (7). However, lung injury and fibrosis are different from BO. The main damaged site is the parenchymal interstitium of the lung in the former, but bronchioles in the latter (7). This phenomenon is in part due to the under-recognition of the disease in children. Also, differences in the clinical phenotypes of COVID-19 pneumonia between adults and children contribute to this disparity. In fact, virus infection-induced lung injury and fibrosis only began receiving enough attention just after the outbreak of SARS-CoV-2 in 2019 (1). Lung injury and fibrosis mainly occurred in cases with severe COVID-19 pneumonia (6). However, severe COVID-19 pneumonia mainly developed in adults rather than children (8).

Viruses such as COVID-19 and influenza are relatively common viruses that have been reported to induce lung injury and fibrosis (1, 5, 6, 9, 10). In addition, herpesvirus-like CMV can also induce or worsen lung injury and fibrosis in cases with immunodeficiency or other injurious stimuli (1, 11). In this study, case 1 was caused by the influenza virus, and case 2 was caused by CMV. Notably, case 3 was caused by HPIV, which has rarely been reported previously. This is the first report of HPIV-induced lung injury and fibrosis. All cases in this study were immunocompromised subjects receiving immunosuppressive therapy.

Virus infection may induce pulmonary fibrosis through two pathways (1). Firstly, it can cause direct damage to the lungs and result in abnormal wound-healing (1). Secondly, it can cause immune-mediated injury (1). Moreover, the courses in all the cases in this study were long. Lung injury and fibrosis have existed to varying degrees before virus infection. Some studies have shown that alveolar epithelial cells of lung fibrosis patients were more susceptible to severe virus-induced injury (12).

Virus infection-induced exacerbations of lung injury and fibrosis should be differentiated from virus infection-induced episodes of alveolar hemorrhage in DAH. Virus infection is also a trigger for the episode of alveolar hemorrhage in the DAH. Besides dyspnea, episodes of alveolar hemorrhage usually present with hemoptysis, a decrease in Hb level, and hemorrhagic BALF. Cases 1 and 2 in this study showed the absence of manifestations of an episode of alveolar hemorrhage mentioned above. As case 3 had a significant decrease in Hb, we considered that she developed an episode of alveolar hemorrhage. However, the abnormality on chest CT in case 3 still existed for longer than the duration of alveolar hemorrhage absorption. It demonstrated that case 3 also developed lung injury and fibrosis. In fact, episodes of alveolar hemorrhage can also result in lung injury and fibrosis (2, 13). Therefore, virus infection-induced exacerbations of lung injury and fibrosis may be more severe in patients with episodes of alveolar hemorrhage in DAH.

Usually, lung fibrosis is referred to as the end stage of a broad range of ILD (1). However, the prognosis of virus infection-induced lung injury and fibrosis varies. In adults, some die eventually, while some get a favorable prognosis (1). In this study, the lesions were partly absorbed in cases 1 and 2, though this process took several months. It demonstrated that virus infection-induced exacerbations of lung injury and fibrosis in the cases with DAH are partially reversible. Some studies demonstrated that administration of anti-fibrotic agents such as nintedanib might be effective for virus infection-induced lung injury and fibrosis (14). In this study, case 1 had taken nintedanib. However, this is just a case report, and more studies are needed to confirm the efficacy and safety of nintedanib for virus infection-induced lung injury and fibrosis in children with DAH.

## Conclusion

Virus infection-induced lung injury and fibrosis should be paid to identification in pediatric DAH. It is partially reversible but may take a long time to recover. NGS is an efficient method for the detection of viruses and should be timely performed in these cases.

## Data Availability

The original contributions presented in the study are included in the article/Supplementary Material; further inquiries can be directed to the corresponding author.
